# Impact of non-motor fluctuations on QOL in patients with Parkinson’s disease

**DOI:** 10.3389/fneur.2023.1149615

**Published:** 2023-04-17

**Authors:** Asako Kakimoto, Miki Kawazoe, Kanako Kurihara, Takayasu Mishima, Yoshio Tsuboi

**Affiliations:** ^1^Department of Neurology, Faculty of Medicine, Fukuoka University, Fukuoka, Japan; ^2^Department of Neurology, Konishi Daiichi Hospital, Fukuoka, Japan; ^3^Department of Preventive Medicine and Public Health, Fukuoka University, Fukuoka, Japan

**Keywords:** Parkinson’s disease, wearing-off phenomenon, motor fluctuation, non-motor fluctuation, wearing-off questionnaire (WOQ-9), Parkinson’s disease questionnaire-8 (PDQ-8)

## Abstract

**Introduction:**

Long-term levodopa treatment in patients with Parkinson’s disease (PwPD) often causes motor fluctuations, which are known to affect their quality of life (QOL). These motor fluctuations may be accompanied by fluctuations in non-motor symptoms. There is no consensus on how non-motor fluctuations affect QOL.

**Methods:**

This was a single-center, retrospective study and included 375 patients with Parkinson’s disease (PwPD) who visited the neurology outpatient department of Fukuoka University Hospital between July 2015 and June 2018. All patients were evaluated for age, sex, disease duration, body weight, and motor symptoms by the Movement Disorder Society-Unified Parkinson’s Disease Rating Scale part III, depression scale by the Zung self-rating depression scale, apathy scale, and cognitive function by the Japanese version of The Montreal Cognitive Assessment. A nine-item wearing-off questionnaire (WOQ-9) was used to assess the motor and non-motor fluctuations. QOL in PwPD was investigated using the eight-item Parkinson’s Disease Questionnaire (PDQ-8).

**Results:**

In total, 375 PwPD were enrolled and classified into three groups according to the presence or absence of motor and non-motor fluctuations. The first group included 98 (26.1%) patients with non-motor fluctuations (NFL group), the second group included 128 (34.1%) patients who presented with only motor fluctuations (MFL group), and the third group included 149 (39.7%) patients without fluctuations in motor or non-motor symptoms (NoFL group). Among them, the PDQ-8 SUM and SI were significantly higher in the NFL group than in the other groups (*p* < 0.005), implying that the NFL group had the poorest QOL among groups. Next, multivariable analysis showed that even one non-motor fluctuation was an independent factor that worsened QOL (*p* < 0.001).

**Conclusion:**

This study showed that PwPD with non-motor fluctuation had a lower QOL than those with no or only motor fluctuation. Moreover, the data showed that PDQ-8 scores were significantly reduced even with only one non-motor fluctuation.

## Introduction

Parkinson’s disease (PD) is characterized by motor symptoms such as tremor, rigidity, and bradykinesia, and a variety of non-motor symptoms such as cognitive impairment, neuropsychiatric symptoms, gastrointestinal symptoms, autonomic dysfunction, pain, and fatigue (1). Advances in diagnosis and treatment for PD have progressed due to the development of recent diagnostic criteria, dopaminergic treatment, and device-aided therapy, and the average life expectancy of patients with Parkinson’s disease (PwPD) has significantly increased (2–4).

As a result, many patients are living with the disease for a longer period of time, and under these circumstances, it is desirable to improve their quality of life (QOL) during that period. Although levodopa remains the most effective therapeutic agent for symptomatic treatment of PD (5, 6), patients often experience motor fluctuations after long-term treatment with levodopa that affect their QOL (7). PwPD with motor fluctuations may also experience fluctuations in their non-motor symptoms (8). Non-motor symptoms are expected to have a greater potential for affecting QOL than motor symptoms in PwPD (9–11). However, there are few studies to date that address how non-motor fluctuations affect patients’ QOL, compared to motor fluctuations. There is no established assessment of non-motor fluctuations except the newly developed MDS-NMS Non-Motor Fluctuations subscale (12). Here, we used the nine-item wearing-off questionnaire (WOQ-9) to evaluate motor and non-motor fluctuations. WOQ-9, which consists of five questions relating to motor symptoms and four questions relating to nonmotor symptoms, was developed as a screening tool for wearing-off, and previous studies propose its efficacy for the early detection of wearing-off (13–15). This study aimed to investigate the impact of non-motor fluctuations for QOL in PwPD using WOQ-9.

## Materials and methods

### Protocol approval

This study was approved by the institutional ethics committee at the Department of Neurology, Fukuoka University Hospital (U20-04-001). Oral, informed consent was obtained from each patient before enrolment and participation in the study.

### Patients and study design

This was a single center, cross sectional, retrospective study of 375 consecutive PwPD. Movement Disorder Society Clinical Diagnostic Criteria for PD (16) were used to diagnose PD, and those patients that met the diagnostic criteria for definite or probable PD were included. All patients enrolled in this study between July 2015 and June 2018 at the Department of Neurology, Fukuoka University Hospital in Japan. All patients were evaluated for age, sex, disease duration, body weight, presence of wearing off, dyskinesia, REM sleep behavior disorder, and visual hallucinations; this information was extracted from each patient’s medical record. Disease severity was defined according to the Hoehn & Yahr stage, and motor symptoms were evaluated using the Movement Disorder Society-Unified Parkinson’s Disease Rating Scale (MDS-UPDRS) part III (17). Cognitive function was assessed with the Japanese version of the Montreal Cognitive Assessment (MOCA-J) (18, 19). Mood disorders were evaluated by the Zung self-rating depression scale (SDS) (20). The evaluation of motor and non-motor fluctuations was performed using WOQ-9, then all patients were classified into three groups according to the results of WOQ-9 as follows: patients with non-motor fluctuations (NFL group), those with only motor fluctuations (MFL group), and those with no fluctuation (NoFL group). Exclusion criteria included dementia, severe psychiatric symptoms, and those not willing to take part in this study. Each patient’s QOL was evaluated by the Parkinson’s Disease Questionnaire-8 (PDQ-8). PDQ-8 is a questionnaire which is a short-form version of the 39-item Parkinson’s Disease Questionnaire (PDQ-39) (21), and the total score (PDQ-8 SUM) and summary index (PDQ-8 SI) were calculated. Then, we studied the correlation between non-motor fluctuations and QOL.

### Statistics

All basic data were expressed as mean ± SD or *n* (%). These data were compared using ANOVA for continuous variables or using chi-square test for categorical variables. This study involved three sets of analyses: In the first, we compared the PDQ-8 SUM/PDQ-8 SI among each of three groups according to the results of WOQ-9 mentioned above. We performed the analysis using analysis of covariance (ANCOVA) with Tukey’s *post-hoc* test, including age, sex, disease duration, MDS-UPDRS part III score, and hallucination as covariates. In the second set of analyses, we assessed associations between the PDQ-8 SUM/PDQ-8 SI and the numbers of motor/non-motor fluctuations in WOQ-9. Additionally, we assessed whether the sum of the numbers of motor/non-motor symptoms had a statistical trend for the PDQ-8 scores by regarding the sum of the numbers as a numerous variable. We performed these analyses using ANCOVA including age, sex, disease duration, and MDS-UPDRS part III score as covariates. In the third set of analyses, we compared the PDQ-8 SUM/PDQ-8 SI among each of four groups divided by disease duration as follows: patients with <2 years duration (DU1 group), those with >2 and < 5 years duration (DU2 group), those with >5 and <10 years duration (DU3 group), and patients with >10 years duration (DU4 group). We performed the analysis using ANCOVA, including age, sex, and MDS-UPDRS part III score as covariates. In the fourth set of analyses, we compared with the item of the non-motor fluctuation among each of motor subtypes of the patients and analyzed the relationship between them by chi-square test. Statistical significance was set at *p* < 0.05. All analyses were conducted using IBM SPSS v.26 and SAS software v.9.4.

## Results

A total of 375 PwPD participated in this study. The first group included 98 (26.1%) patients with non-motor fluctuations (NFL group), the second group included 128 (34.1%) patients who presented with only motor fluctuations (MFL group), and the third group included 149 (39.7%) patients without fluctuations in motor or non-motor symptoms (NoFL group). The demographics and clinical characteristics among the three groups are presented in Table 1. The age, and age at PD onset of the NoFL group were significantly higher than those of the other two groups. The disease duration of the NoFL group was shorter than those of the other two groups (*p* = 0.038). The higher scores of PDQ-8 corresponded to lower scores of QOL (Table 1).

**Table 1:** Demographics and clinical characteristics.

	Total (*n* = 375)	NFL (*n* = 98, M + NF = 93, NF = 5)	MFL (*n* = 128)	NoFL (*n* = 149)	Value of *p*
Sex, male, *n* (%)	140 (37.3%)	33 (33.7%)	33 (25.8%)	74 (49.7%)	< 0.001
Age, year (SD)	69.8 (10.6)	67.7 (11.0)	68.8 (11.8)	72.0 (8.8)	0.004
Age at onset, year (SD)	62.1 (11.8)	58.9 (12.1)	60.9 (12.0)	65.1 (10.8)	< 0.001
Disease duration, year (SD)	7.8 (6.4)	8.9 (4.7)	8.1 (5.9)	6.9 (7.5)	0.041
RBD, *n* (%)	177 (47.8%)	58 (59.8%)	55 (44.0%)	64 (43.2%)	0.022
Hallucinations, *n* (%)	97 (26.0%)	36 (36.7%)	26 (20.6%)	35 (23.5%)	0.019
H&Y	2.8 (1.8)	2.8 (0.8)	2.7 (0.9)	2.9 (2.7)	0.733
MDS-UPDRS part III	30.4 (14.7)	31.7 (15.5)	30.0 (15.8)	30.0 (12.9)	0.645
Motor fluctuation number (SD)	1.2 (1.3)	2.3 (1.2)	1.8 (0.9)	0	< 0.001
Non-motor fluctuation number (SD)	0.5 (0.9)	1.7 (0.8)	0	0	< 0.001
MMSE (SD)	26.9 (3.3)	26.7 (3.6)	27.5 (3.2)	26.8 (3.2)	0.092
MoCA (SD)	22.7 (3.4)	22.4 (5.7)	23.4 (4.8)	22.2 (4.3)	0.123
PDQ-8 SI (SD)	21.7 (19.3)	29.7 (20.1)	20.6 (20.6)	17.4 (15.1)	< 0.001
PDQ-8 SUM (SD)	6.9 (6.2)	9.5 (6.7)	6.6 (6.6)	5.6 (4.8)	< 0.001
SDS (SD)	43.0 (10.3)	46.7 (9.7)	42.2 (10.0)	41.2 (10.3)	0.001

NFL, Patients with non-motor fluctuations; MFL, Patients with only motor fluctuations; NoFL, Patients with no fluctuations; M + NF, Patients with non-motor fluctuations and motor fluctuations; PD, Parkinson disease; RBD, REM sleep behavior disorder; H&Y, Hoehn & Yahr scale; MDS-UPDRS, Movement Disorder Society-Unified Parkinson’s Disease Rating Scale; MMSE, Mini-Mental State Examination; MoCA, The Montreal Cognitive Assessment; PDQ-8 SI, The Parkinson’s Disease Questionnaire-8 summary index; PDQ-8 SUM, The Parkinson’s Disease Questionnaire-8 sum score; WOQ-9, 9-item Wearing-off Questionnaire; SDS: self-rating depression scale.

In the first analysis, the scores of PDQ-8 SUM and PDQ-8 SI in the NFL group were significantly higher than those in the other two groups even after adjusting for covariates (NFL group vs. MFL group regarding PDQ-8 SUM: differences, 2.5, 95% CI 1.0–4.0, value of *p*, 0.003; NFL group vs. NoFL group regarding PDQ-8 SUM: differences, 3.0, 95% CI, 1.5–4.5, value of *p* < 0.001; NFL group vs. MFL group regarding PDQ-8 SI: differences, 7.8, 95% CI, 3.2–12.4, value of *p*, 0.003; NFL group vs. NoFL group regarding PDQ-8 SI: differences 9.3, 95% CI, 4.7–14.0, value of *p* < 0.001; Table 2).

**Table 2:** Multivariable analysis of the association between PDQ-8 SUM/PDQ-8 SI and the three groups according to the results of WOQ-9.

	NFL vs. MFL	NFL vs. NoFL	MFL vs. NoFL
PDQ-8 SI	NFLMFL	NFLNoFL	MFLNoFL
Mean (95% CI)	28.1(24.5–31.6), 20.3 (17.2–23.4)	28.1(24.5–31.6), 18.7 (15.9–21.6)	20.3 (17.2–23.4), 18.7 (15.9–21.6)
Difference	7.8 (3.2–12.4)	9.3 (4.7–14.0)	1.6 (−2.7–5.8)
*p* value	0.003	<0.001	0.751
PDQ-8 SUM	NFLMFL	NFLNoFL	MFLNoFL
Mean (95% CI)	9.0 (7.8–10.1), 6.5 (5.5–7.5)	9.0 (7.8–10.1), 6.0 (5.1–6.9)	6.5 (5.5–7.5), 6.0 (5.1–6.9)
Difference	2.5 (1.0–4.0)	3.0 (1.5–4.5)	0.5 (−0.9–1.9)
*p* value	0.003	<0.001	0.752

NFL, Patients with non-motor fluctuations; MFL, Patients with only motor fluctuations; NoFL, Patients with no fluctuations; PDQ-8 SI, The Parkinson’s Disease Questionnaire-8 summary index; PDQ-8 SUM, The Parkinson’s Disease Questionnaire-8 sum score; WOQ-9, 9-item Wearing-off Questionnaire; CI, confidence interval. For multivariable analysis, mean differences, 95% confidence intervals, and value of *p*s were estimated using ANCOVA with Tukey’s *post hoc* test, including age, sex, disease duration, MDS-UPDRS part III score, and hallucination as covariates.

**Figure 1:**
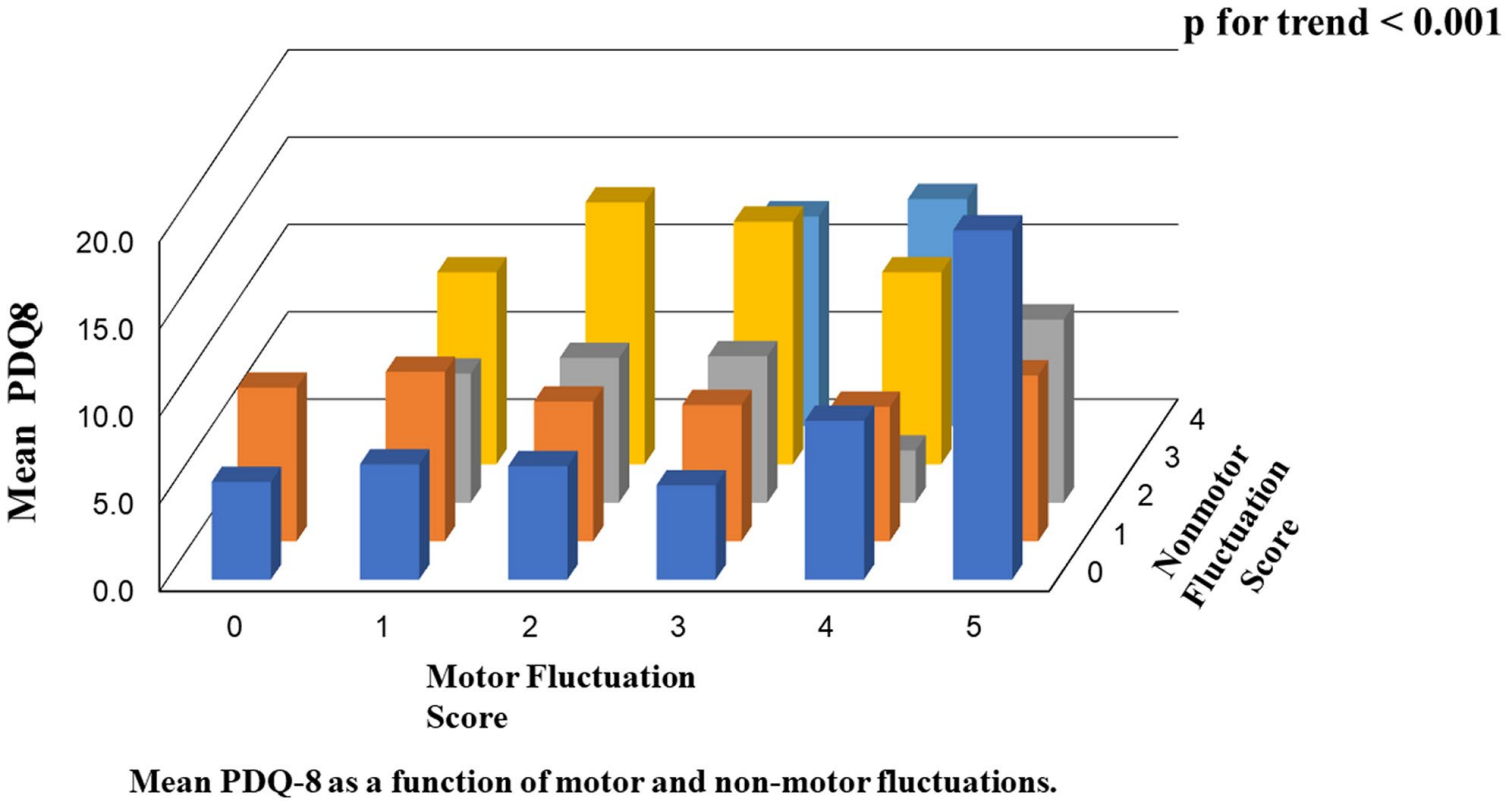
Motor and nonmotor fluctuations.

In the second analysis, there were linear associations between the scores of PDQ-8 SUM/PDQ-8 SI and the numbers of motor/non-motor fluctuations. The value of *p* trend for the associations between motor numbers and PDQ-8 SUM, motor numbers and PDQ-8 SI, non-motor numbers and PDQ-8 SUM, and non-motor numbers and PDQ-8 SI were 0.003, 0.003, <0.001, and < 0.001, respectively. Furthermore, the value of *p* trend for the associations between the total number of motor and non-motor fluctuations and PDQ-8 SUM and between the total number of motor and non-motor fluctuations and PDQ-8 SI were <0.001 and <0.001, respectively (Table 3; Figure 1).

**Table 3:** Relationship between the scores of PDQ-8 SUM/PDQ-8 SI and the number of motor fluctuations; relationship between the scores of PDQ-8 SUM/ PDQ-8 SI and the number of non-motor fluctuations.

	The numbers of motor fluctuation						*p* for trend
	0	1	2	3	4	5	
*N*	154	77	79	40	20	5	
PDQ-8 SI Mean (SD)	17.7 (15.2)	22.6 (21.5)	23.6 (21.0)	26.5 (22.7)	27.7 (20.1)	37.5 (16.1)	0.003
PDQ-8 SUM Mean (SD)	5.7 (4.9)	7.2 (6.9)	7.6 (6.7)	8.5 (7.3)	8.9 (6.4)	12.0 (5.2)	0.003
	The numbers of non-motor fluctuation						*p* for trend
	0	1	2	3	4		
N	277	51	26	19	2		
PDQ-8 SI Mean (SD)	18.9 (17.9)	27.9 (20.6)	25.1 (20.4)	39.8 (21.2)	39.1 (2.2)		<0.001
PDQ-8 SUM Mean (SD)	6.0 (5.7)	8.9 (6.6)	8.0 (6.5)	12.7 (6.8)	12.5 (0.7)		<0.001

PDQ-8 SI, The Parkinson’s Disease Questionnaire-8 summary index; PDQ-8 SUM, The Parkinson’s Disease Questionnaire-8 sum score; WOQ-9, 9-item Wearing-off Questionnaire.

Among the items in WOQ-9, pain is the most weighted fluctuated symptom related to QOL, followed by anxiety. In addition, a multivariable analysis was performed to examine the association between combination of WOQ-9 items on non-motor fluctuation and QOL, but no combination was found to be correlated (*p* = 0.157).

**Figure 2:**
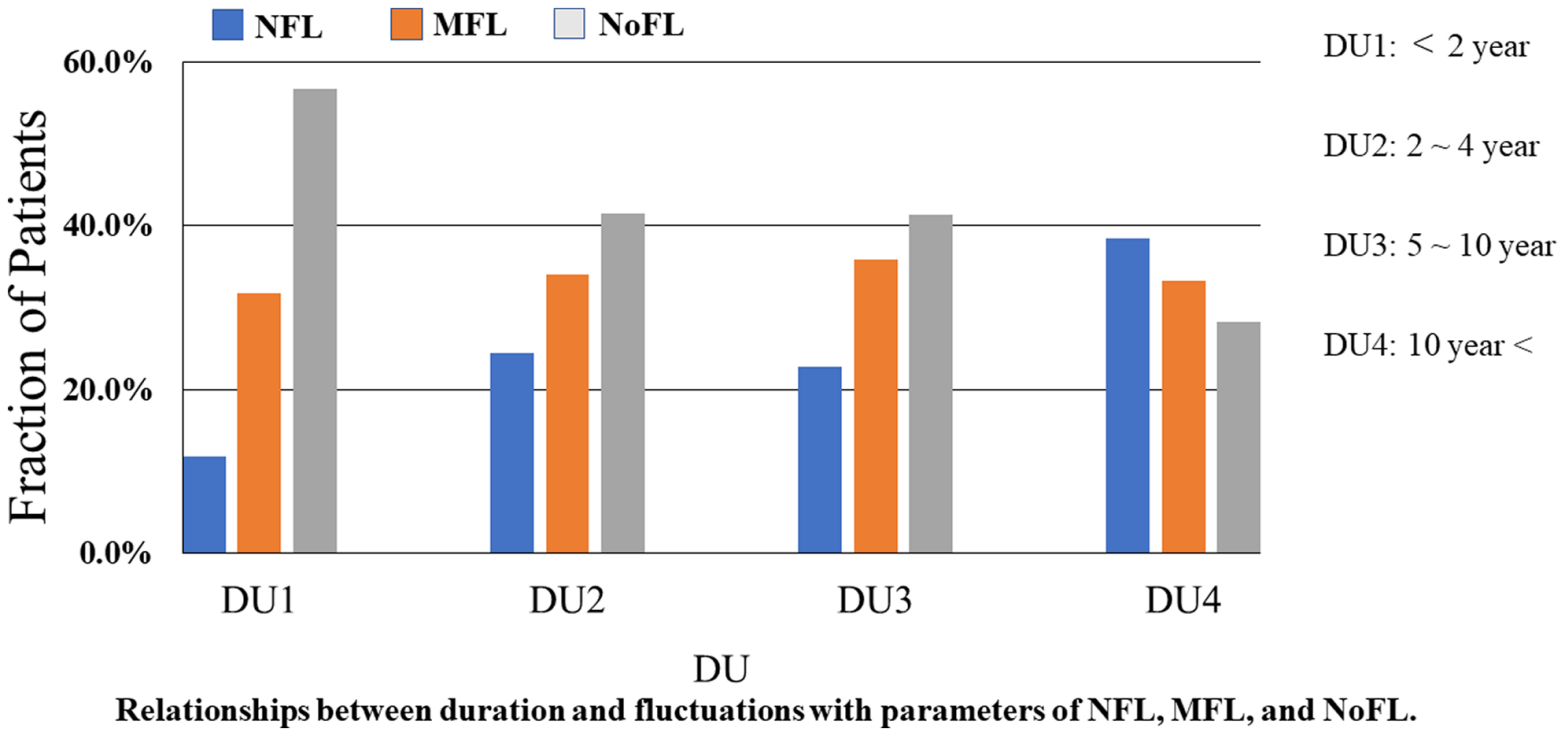
Relationship between duration and fluctuations.

In the third analysis, all of the 375 PwPD were classified by disease duration into the following four groups. We used ANCOVA including age, sex, and MDS-UPDRS part III score as covariates. The first group which contained 60 (16.0%) patients was classified as a DU1. The second group had 53 (14.1%) patients was labeled DU2. The third group labeled as DU3 had 145 (38.7%) patients. Then, the fourth group, DU4, had 117 (31.2%) patients. We studied the relationships between disease duration and fluctuations in PwPD (Figure 2). The proportion of PwPD with motor and non-motor fluctuations increased when the PD disease duration increased (*p* < 0.05).

In the fourth analysis, two out of the 375 PwPD were excluded due to lack of motor subtype data, then the total of 373 PwPD was classified by motor subtypes into the three groups: tremor-dominant, postural instability and gait difficulty, and mixed. The non-motor fluctuations of mood changes, pain, cloudy/thinking, and anxiety/panic among the three groups were analyzed by chi-square test. We obtained a clear relationship between the non-motor fluctuation of cloudy/thinking and motor subtypes (*p* < 0.05), while others have no relationship with motor subtypes (Table 4). PwPD with the motor subtype of postural instability and gait difficulty have a clear correlation with the non-motor fluctuation of cloudy/thinking.

**Table 4:** Mood changes between PwPD with the three motor subtypes according to the results of WOQ-9; pain/aching between PwPD with the three motor subtypes according to the results of WOQ-9; cloudy/thinking between PwPD with the three motor subtypes according to the results of WOQ-9; and anxiety/Panic between PwPD with the three motor subtypes according to the results of WOQ-9.

		Motor subtypes			*p* value
WOQ-9		Tremor-dominant	Postural instability and gait difficulty	Mixed	
Mood changes	Yes	22 (5.9)	26 (7.0)	4 (1.1)	0.181
	None	180 (48.3)	122 (32.7)	19 (5.1)	
Pain/Aching	Yes	18 (4.8)	18 (4.8)	2 (0.5)	0.593
	None	184 (49.3)	130 (34.9)	22 (5.9)	
Cloudy/Thinking	Yes	17 (4.6)	30 (8.0)	2 (0.5)	0.004
	None	185 (49.6)	118 (31.6)	21 (5.6)	
Anxiety/Panic	Yes	11 (2.9)	14 (3.8)	4 (1.1)	0.079
	None	191 (51.2)	134 (35.9)	19 (5.1)	

WOQ-9, 9-item Wearing-off Questionnaire. In the four present analyses, *p* values were estimated using chi-square tests. Statistical significance was defined as value of *p* < 0.05. All data are presented as *n* (%).

## Discussion

This single-center, retrospective study conducted in Japan showed that non-motor fluctuations were an independent risk factor for reducing patients’ QOL. Prevalence of non-motor fluctuations were found in 26.1% of the participants, and increased with severity of PD. These results were slightly lower overall than the frequency of NMF in the MDS-NMS study of 9.1% for <2 years of disease duration, 54.3% for 2–5 years, 63.6% for 5–10 years, and 71.0% for ≥10 years, with an average of 49.2% (22). It is known that factors such as older age, disease duration, reduced activity of daily living, severity of motor symptoms, and long off-time reduce the QOL score of PwPD (23). Using a score that quantified non-motor symptoms, it has been reported that non-motor symptoms are more important than motor symptoms for QOL in PwPD (11). To date, no studies have evaluated the relationship between non-motor fluctuations and QOL in comparison with motor fluctuations. In this study, PDQ-8 scores such as SUM and SI in the NFL group were higher than scores for MFL and NoFL (*p* < 0.001), implying that the NFL group had the poorest QOL among groups. WOQ-9 is a simple tool that can be easily used in daily clinical practice for research, as opposed to a complicated questionnaire. It was shown that non-motor fluctuations obtained in this assessment directly affected QOL, making it a target for more aggressive therapeutic intervention. Generally, non-motor fluctuations appear after the presence of motor fluctuations, however, in this study there were a few cases in which only non-motor fluctuations were seen, without the motor fluctuations shown in the previous study (10). In addition, it was observed that non-motor fluctuations could appear even at an early stage; alternately, there were many patients who continued with only motor fluctuations for a long time. Thus, it can be considered that the group with the earliest occurrence of non-motor fluctuations formed a subtype with poor QOL such as a subtype with severe motor and non-motor dysfunction/malignancy.

This study has some limitations. This was a single center, cross-sectional study, therefore the number of patients was limited. However, reliability of the data was confirmed because diagnosis and clinical evaluation was performed by specialists of movement disorders. Patients were consecutive without selection, and they were involved from an early stage to a progressive one. Second, the study did not include patients with severe dementia or more advanced stages. Third, the study did not include detailed information on medications other than LED. Medication content may affect the prevalence of non-motor fluctuations, and further investigation considering the type of antiparkinsonian medications is necessary. Fourth, evaluation of non-motor fluctuations by MDS-NMS, which has recently been validated as a new qualitative test, was not performed in this study. In addition, the reason for the lower non-motor fluctuation prevalence compared to the MDS-NMS Non-Motor Fluctuations subscale (12) in this study may have been due to the lower sensitivity of non-motor fluctuations as a result of using WOQ-9 rather than MDS-NMS. However, the usefulness of WOQ-9 lies in its simplicity, which makes it suitable for use during routine clinical practice. We here investigated how combinations within the four non-motor items of WOQ-9 correlated with QOL, but owing to the small number of patients in each group, we were unable to obtain significant differences. Since it seems important to ascertain which combinations of non-motor symptom items are most relevant to quality of life, future large-scale studies should be conducted to clarify this point. Further research is also needed to analyze the risk factors for non-motor fluctuations, which could not be examined here.

In conclusion, this is the first report to assess the prevalence of non-motor fluctuations using a simplified WOQ-9 in PwPD. Furthermore, it was shown that the non-motor fluctuations affected QOL independently of motor fluctuations. Non-motor fluctuations should therefore be accurately evaluated in PwPD.

## Data availability statement

The original contributions presented in the study are included in the article/supplementary material, further inquiries can be directed to the corresponding author.

## Ethics statement

The studies involving human participants were reviewed and approved by Institutional ethics committee at the Department of Neurology, Fukuoka University Hospital. Written informed consent for participation was not required for this study in accordance with the national legislation and the institutional requirements.

## Author contributions

AK: methodology, statistical analysis, investigation, and writing—original draft. MK: statistical analysis. KK and TM: review and editing. YT: conceptualization, formal analysis, and supervision. All authors contributed to the article and approved the final version.

## Conflict of interest

The authors declare that the research was conducted in the absence of any commercial or financial relationships that could be construed as a potential conflict of interest.

## Publisher’s note

All claims expressed in this article are solely those of the authors and do not necessarily represent those of their affiliated organizations, or those of the publisher, the editors and the reviewers. Any product that may be evaluated in this article, or claim that may be made by its manufacturer, is not guaranteed or endorsed by the publisher.

## References

[1] Sveinbjornsdottir S. The clinical symptoms of Parkinson’s disease. J Neurochem. (2016) 139:318–24. doi: 10.1111/jnc.13691 27401947

[2] Wanneveich M Moisan F Jacqmin-Gadda H Elbaz A Joly P. Projections of prevalence, lifetime risk, and life expectancy of Parkinson’s disease (2010-2030) in France. Mov Disord. (2018) 33:1449–55. doi: 10.1002/mds.27447, PMID: 30145805

[3] Rajput AH Uitti RJ Rajput AH Offord KP. Timely levodopa (LD) administration prolongs survival in Parkinson’s disease. Parkinsonism Relat Disord. (1997) 3:159–65. doi: 10.1016/s1353-8020(97)00030-8, PMID: 18591070

[4] Scigliano G Musicco M Soliveri P Piccolo I Girotti F Giovannini P. Mortality associated with early and late levodopa therapy initiation in Parkinson’s disease. Neurology. (1990) 40:265–9. doi: 10.1212/wnl.40.2.265, PMID: 2300246

[5] Fox SH Katzenschlager R Lim SY Barton B de Bie RMA Seppi K. Movement Disorder Society evidence-based medicine committee. International Parkinson and movement disorder society evidence-based medicine review: update on treatments for the motor symptoms of Parkinson’s disease. Mov Disord. (2018) 33:1248–66. doi: 10.1002/mds.27372, PMID: 29570866

[6] Cilia R Akpalu A Sarfo FS Cham M Amboni M Cereda E. The modern pre-levodopa era of Parkinson’s disease: insights into motor complications from sub-Saharan Africa. Brain. (2014) 137:2731–42. doi: 10.1093/brain/awu195, PMID: 25034897 PMC4163032

[7] Obeso JA Grandas F Vaamonde J Luquin MR Artieda J Lera G. Motor complications associated with chronic levodopa therapy in Parkinson’s disease. Neurology. (1989) 39:11–9. PMID: 2685647

[8] Witjas T Kaphan E Azulay JP Blin O Ceccaldi M Pouget J. Nonmotor fluctuations in Parkinson’s disease: frequent and disabling. Neurology. (2002) 59:408–13. doi: 10.1212/wnl.59.3.408, PMID: 12177375

[9] Pfeiffer RF. Non-motor symptoms in Parkinson’s disease. Parkinsonism Relat Disord. (2016) 22:S119–22. doi: 10.1016/j.parkreldis.2015.09.004 26372623

[10] Chaudhuri KR Healy DG Schapira AH. Non-motor symptoms of Parkinson’s disease: diagnosis and management. Lancet Neurol. (2006) 5:235–45. doi: 10.1016/S1474-4422(06)70373-8 16488379

[11] Martinez-Martin P Rodriguez-Blazquez C Kurtis MM Chaudhuri KR NMSS Validation Group. The impact of non-motor symptoms on health-related quality of life of patients with Parkinson’s disease. Mov Disord. (2011) 26:399–406. doi: 10.1002/mds.23462 21264941

[12] Rodriguez-Blazquez C Schrag A Rizos A Chaudhuri KR Martinez-Martin P Weintraub D. Prevalence of non-motor symptoms and non-motor fluctuations in Parkinson’s disease using the MDS-NMS. Mov Disord Clin Pract. (2021) 8:231–9. doi: 10.1002/mdc3.13122, PMID: 33553493 PMC7853195

[13] Stacy MA Murphy JM Greeley DR Stewart RM Murck H Meng X. The sensitivity and specificity of the 9-item wearing-off questionnaire. Parkinsonism Relat Disord. (2008) 14:205–12. doi: 10.1016/j.parkreldis.2007.07.013, PMID: 17900967

[14] Fukae J Higuchi MA Yanamoto S Fukuhara K Tsugawa J Ouma S. Utility of the Japanese version of the 9-item wearing-off questionnaire. Clin Neurol Neurosurg. (2015) 134:110–5. doi: 10.1016/j.clineuro.2015.04.021, PMID: 25985062

[15] Stacy M Bowron A Guttman M Hauser R Hughes K Larsen JP. Identification of motor and nonmotor wearing-off in Parkinson’s disease: comparison of a patient questionnaire versus a clinician assessment. Mov Disord. (2005) 20:726–33. doi: 10.1002/mds.20383, PMID: 15719426

[16] Postuma RB Berg D Stern M Poewe W Olanow CW Oertel W. MDS clinical diagnostic criteria for Parkinson’s disease. Mov Disord. (2015) 30:1591–601. doi: 10.1002/mds.26424 26474316

[17] Kashihara K Kondo T Mizuno Y Kikuchi S Kuno S Hasegawa K. Official Japanese version of the International Parkinson and Movement Disorder Society-unified Parkinson’s disease rating scale: validation against the original English version. Mov Disord Clin Pract. (2014) 1:200–12. doi: 10.1002/mdc3.12058, PMID: 25328906 PMC4199098

[18] Nasreddine ZS Phillips NA Bedirian V Bédirian V Charbonneau S Whitehead V. The Montreal cognitive assessment, MoCA: a brief screening tool for mild cognitive impairment. J Am Geriatr Soc. (2005) 53:695–9. doi: 10.1111/j.1532-5415.2005.53221.x 15817019

[19] Fujiwara Y Suzuki H Yasunaga M Sugiyama M Ijuin M Sakuma N. Brief screening tool for mild cognitive impairment in older Japanese: validation of the Japanese version of the Montreal cognitive assessment. Geriatr Gerontol Int. (2010) 10:225–32. doi: 10.1111/j.1447-0594.2010.00585.x, PMID: 20141536

[20] Zung WW. A self-rating depression scale. Arch Gen Psychiatry. (1965) 12:63–70. doi: 10.1001/archpsyc.1965.01720310065008 14221692

[21] Peto V Jenkinson C Fitzpatrick R. PDQ-39: a review of the development, validation and application of a Parkinson’s disease quality of life questionnaire and its associated measures. J Neurol. (1998) 245:S10–4. doi: 10.1007/pl00007730, PMID: 9617716

[22] van Wamelen DJ Rota S Schrag A Rizos A Martinez-Martin P Weintraub D. Characterization of non-motor fluctuations using the movement disorder society non-motor rating scale. Mov Disord Clin Pract. (2022) 9:932–40. doi: 10.1002/mdc3.13520, PMID: 36247921 PMC9547143

[23] Zhao N Yang Y Zhang L Zhang Q Balbuena L Ungvari GS. Quality of life in Parkinson’s disease: a systematic review and meta-analysis of comparative studies. CNS Neurosci Ther. (2021) 27:270–9. doi: 10.1111/cns.13549, PMID: 33372386 PMC7871788

